# Systemic lupus erythematosus and risk factors for adverse outcomes in pregnancy: a single center retrospective cohort study in Northern Brazil

**DOI:** 10.1186/s42358-025-00467-2

**Published:** 2025-07-28

**Authors:** Sérgio Henrique Oliveira dos Santos, Juliana Bühring, Luiz Fernando de Souza Passos, Bárbara Seabra Carneiro, Domingos Sávio Nunes de Lima, Sandra Lúcia Euzébio Ribeiro

**Affiliations:** 1 Hospital Universitário Getúlio Vargas, Universidade Federal do Amazonas, R. Tomas de Vila Nova, 300 - Centro, Manaus, Amazonas 69020-170 Brasil; 2 Faculdade de Medicina, Universidade Federal do Amazonas, R. Afonso Pena, 1053 - Centro, Manaus, Amazonas 69020-160 Brasil; 3 Faculdade de Medicina, Universidade do Estado do Amazonas, Av. Carvalho Leal, 1777 - Cachoeirinha, Manaus, Amazonas 69065-001 Brasil

**Keywords:** Systemic lupus erythematosus, Pregnancy, Outcomes, Hydroxychloroquine, Disease activity

## Abstract

**Background:**

Systemic lupus erythematosus (SLE) directly impacts pregnancy outcomes, and few studies have analyzed the related risk factors for maternal and fetal/perinatal complications in Brazil. We described and analyzed the risk factors for maternal and fetal complications in SLE pregnancies at an outpatient clinic in the State of Amazonas, Northern Brazil.

**Methods:**

Pregnancies that occurred after the SLE diagnosis between 2001 and 2020 were analyzed. Risk factors for adverse outcomes were determined using logistic regression.

**Results:**

A total of 155 pregnancies from 109 women were included; the mean age was 28.2 (*±*5.5) years, the median disease duration was 72 [36; 108] months, 56 (36.1%) had active disease prior to pregnancy, 39 (26.5%) had nephritis, 30 (20.3%) had cutaneous manifestations, and the incidence of disease activity during pregnancy was 29.7%; there was a 12.3% (95% CI, [4.2; 20.4]) increase in the proportion of patients with active disease, there were 35 (22.9%) fetal deaths, 26 (16.8%) cases of preeclampsia, 44 (37.3%) preterm births, 16 (16.2%) cases of low birth weight for gestational age, and 18 (18.8%) cases of intrauterine growth restriction. Risk factors for maternal events included immunological alterations (OR=3.02; 95% CI [1.11; 8.21]), renal involvement (OR=3.74; 95% CI [1.25; 11.21]), and disease activity before pregnancy (OR=1.30; 95% CI [1.04; 1.64]); the use of hydroxychloroquine before pregnancy was protective (OR=0.23; 95% CI [0.08; 0.67]). Risk factors for fetal events included the use of acetylsalicylic acid (ASA) during pregnancy (OR=5.22; 95% CI [1.33; 20.54]) and disease activity during pregnancy (OR=1.31; 95% CI [1.14; 1.52]); the use of antimalarials before and during pregnancy was protective (OR=0.14; 95% CI [0.04; 0.44]). According to the post-hoc analysis, the probability of a Type S error in the ASA association was 100%. The retrospective nature and the presence of missing data about laboratory tests are the main limitations of this study.

**Conclusion:**

Pregnancy management in SLE presents a unique set of challenges that require a comprehensive and multidisciplinary approach. Careful monitoring of disease activity, appropriate medication management, and psychosocial support are essential for optimizing maternal and fetal health outcomes.

## Introduction

Systemic lupus erythematosus (SLE) is a multisystemic immune-mediated disease that predominantly affects women of childbearing age. It does not directly interfere with fertility but can lead to serious maternal, fetal, and perinatal complications such as preeclampsia, fetal death, prematurity, and disease activity during and after pregnancy. The likelihood of developing complications during pregnancy is two to four times higher than in the healthy population [1].

Smyth et al. [2] and Bundhun et al. [3] showed that SLE has a strong impact on pregnancy outcomes, both from maternal and fetal perspective. SLE activity, a history of nephritis, presence of antiphospholipid antibodies (aPL), and previous thromboembolic events are risk factors for adverse maternal, fetal, and perinatal events [4–6].

Although SLE and pregnancy are widely studied, there are few studies describing the adverse events of pregnancy in Brazil [7]. We describe and analyze the risk factors for maternal, fetal, and perinatal complications in the pregnancies of women with SLE in the State of Amazonas. Our primary goals were to describe the incidence of maternal, fetal, and perinatal complications in pregnancies of patients with SLE and to determine the risk factors associated with these adverse outcomes.

## Methods

### Study design

This observational study retrospectively described and analyzed the pregnancies of patients who met the classification criteria for SLE according to the 1997 revised American College of Rheumatology (ACR) criteria [8]. The study covered the period from 2001 to 2020 and included patients who had at least one pregnancy after diagnosis and were in regular follow-up until one year before the initial pregnancy consultation. Patients with multiple fetal pregnancies, nontherapeutic abortions, or those under 18 years of age at the time of the study were excluded. Data were obtained from the medical records of patients followed at the Araújo Lima outpatient clinic of the Federal University of Amazonas.

### Sample size estimation

The sample size estimation was calculated using Fleiss’ method with continuity correction, a significance level of 95%, a statistical power of 80%, and based on Clowse’s study [9], which investigated the effect of SLE activity on maternal, fetal, and perinatal complications. Due to the possibility of missing data, we arbitrarily estimated that an additional approximately 20% would be necessary, totaling at least 76 pregnancies.

### Variable definition

The maternal demographic variables included age in years at the first rheumatologist visit where pregnancy was reported, disease duration in months, past medical history, medication use, autoantibody profile, number of visits and gestational age in weeks (measured by the date of the last menstrual period or first trimester ultrasound when available in medical record).

Data prior to pregnancy, medications and SLE activity, were obtained from consultations up to 12 months before the first pregnancy consultation. Data regarding the gestational period were obtained from the first and last consultations before delivery, and data on activity and medications post-delivery were collected from the first consultation after birth up to the consultation closest to 12 months after that date.

Disease activity was assessed using the Systemic Lupus Erythematosus Disease Activity Index (SLEDAI) [10]. SLE flare was defined as the disease becoming active during the analyzed period after being in remission in the preceding one. Worsening of SLE corresponded to a positive variation in the SLEDAI score in the analyzed period compared to the preceding one.

The adverse maternal outcomes evaluated included preeclampsia, gestational loss, hypertension during pregnancy (systolic BP*≥* 140 mmHg and diastolic BP*≥* 90 mmHg), and exacerbation or worsening of the disease during and after pregnancy.

The fetal and perinatal outcomes considered were fetal death: spontaneous abortion (gestational age (GA)*≤* 20 weeks) or stillbirth (GA* > * 20 weeks), prematurity (GA* < * 37 weeks), low birth weight for gestational age (below the 10th percentile for GA), and intrauterine growth restriction (IUGR): recorded in medical records or as defined by reference values from studies of Mandy [11], Napolitano et al. [12], Villar et al. [13].

### Statistical analysis

The data were analyzed using the Statistical Package for the Social Sciences (SPSS) version 20, with two-tailed statistical tests, a type I error probability set at 5%, and a 95% confidence interval for all tests.

Categorical data are presented as absolute and relative frequencies in parentheses; continuous data, depending on their distribution as assessed by the Shapiro-Wilk test, are presented as mean with standard deviation are shown in parentheses or medians with interquartile ranges are shown in brackets.

Nominal comparisons were made using the chi–square test, Fisher’s exact test, or McNemar’s test for paired analysis. Continuous comparisons were made with the Mann–Whitney or Student’s t–test, or Wilcoxon or paired Student’s t–test.

Logistic regression (LR) was used to adjust the odds ratio (OR) for associations with an *α* of up to 0.25; multicollinearity was assessed, and variables with a Variance Inflation Factor (VIF) * > * 2.5 [14–19] were removed from the models.

To evaluate predictors, LR was performed using the Forward Stepwise Logistic Regression method, with an entry *α* set at 0.05 and an exit *α* set at 0.1; the model adjustment quality was assessed with the Hosmer and Lemeshow test.

Missing data did not undergo imputation processes and were excluded from each statistical analysis. The sample size (N) for each analysis corresponds to the number of valid records, which are reported in the text, tables, and figures where applicable.

## Results

### Inclusion, clinical characteristics and demographic data

**Fig. 1:**
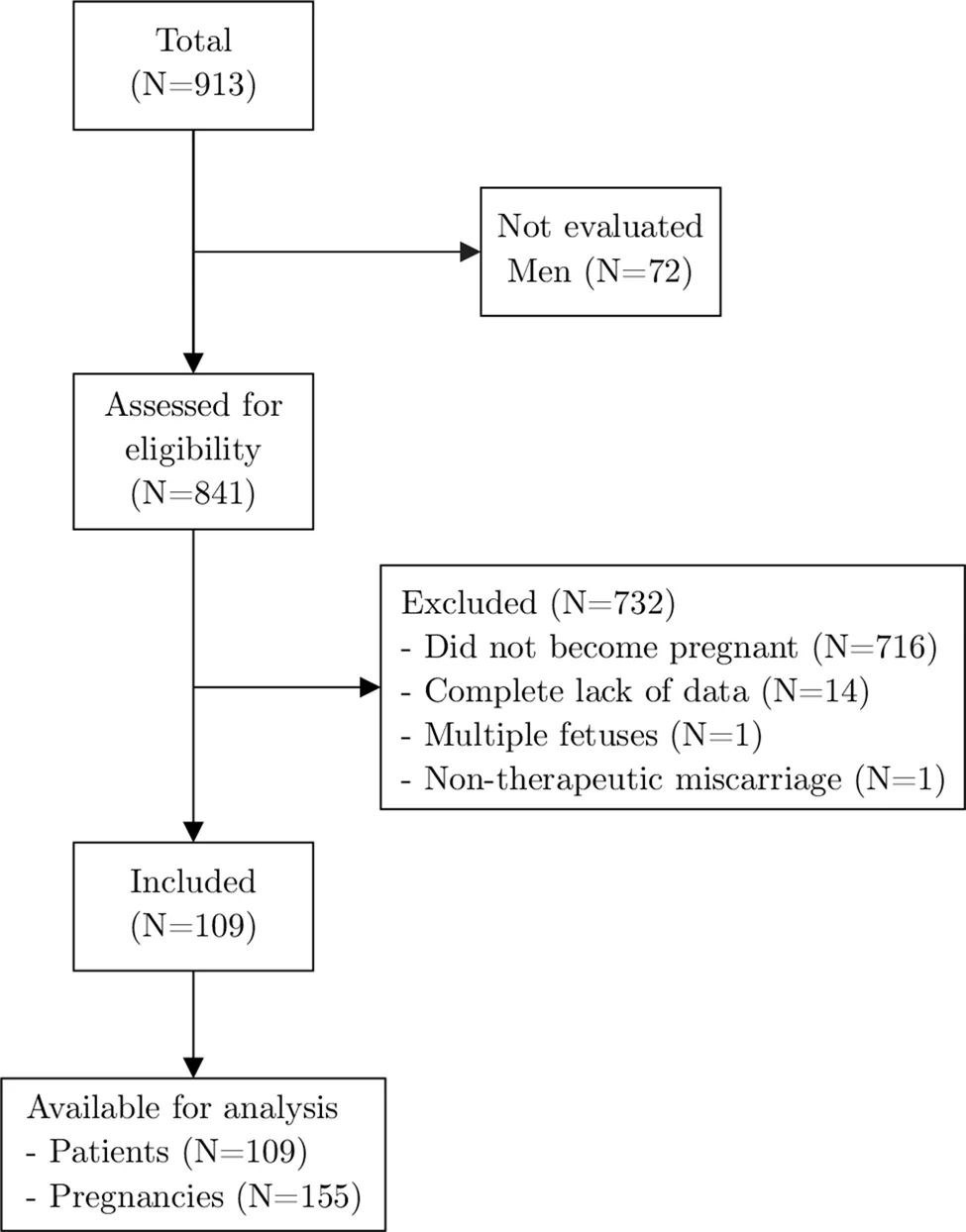
Flowchart describing the number of records obtained in the eligibility assessment, inclusion, and those available for analysis

A total of 913 medical records of patients with SLE were reviewed, 841 (92.1%) were assessed for eligibility, and 109 (11.9%) were included, with a total of 155 pregnancies Fig. 1. The median disease duration was 72 months [36; 108], and the mean age at the time of pregnancy was 28.2 years (*±*5.5); 94 (60.6%) patients had at least one comorbidity. The median number of consultations during pregnancy was 4 [2; 5]. The distribution of the ACR/97 criteria, autoantibody profile, and comorbidities are described in Table 1.

**Table 1:** Demographic and clinical characteristics of the 155 pregnancies in 109 patients with SLE

Characteristics		N$^1$
Maternal age$^2$, mean (*±* standard deviation)	28.2 (*±*5.5)	155
Disease duration$^2$, median [P25; P75]	72 [36; 108]	155
Gestational age$^2$, median [P25; P75]	10 [7; 15]	141
Number of consultations$^2$, median [P25; P75]	4 [2; 5]	144
Photosensitivity, n (%)	129 (83.2)	155
Malar rash, n (%)	111 (71.6)	155
Discoid rash, n (%)	12 (7.7)	155
Oral ulcer, n (%)	56 (36.1)	155
Arthritis, n (%)	138 (89.0)	155
Serositis, n (%)	51 (32.9)	155
ANA, n (%)	150 (96.8)	155
Immunological disorder, n (%)	93 (60.0)	155
Neurological disorder, n (%)	15 (9.7)	155
Hematological disorder, n (%)	80 (51.6)	155
Renal disorder, n (%)	76 (49.0)	155
Anti-double stranded DNA, n (%)	75 (48.4)	155
Anti-SM, n (%)	15 (9.7)	155
Anti-RNP, n (%)	4 (2.6)	155
Anti-SSA, n (%)	38 (24.5)	155
Anti-SSB, n (%)	8 (5.2)	155
Anticardiolipin, n (%)	34 (21.9)	155
Lupus anticoagulant, n (%)	11 (7.1)	155
Comorbidities, n (%)	94 (60.6)	155
Chronic kidney disease, n (%)	50 (32.3)	155
Systemic arterial hypertension, n (%)	45 (29.0)	155
Dyslipidemia, n (%)	19 (12.3)	155
Diabetes, n (%)	4 (2.6)	155
Antiphospholipid syndrome (APS), n (%)	18 (11.6)	155
Aseptic osteonecrosis, n (%)	7 (4.5)	155
Previous thromboembolic event, n (%)	16 (10.3)	155

^1^Valid N. The difference between the total N of 155 and the valid N corresponds to the missing data.

^2^Notes: Maternal age at the time of pregnancy (years), disease duration (months), gestational age at the first consultation (weeks), number of consultations during pregnancy

### Prepregnancy period

In the period prior to pregnancy, SLE was in remission in 91 pregnancies (61.9%), while it was active in 56 pregnancies (38.1%) with a median SLEDAI of 6 [4; 8]. Among the active cases, 39 (69.6%) had nephritis, 30 (53.5%) had cutaneous activity, 26 (46.4%) had hematological disorders, and 18 (32.1%) had arthritis. Immunosuppressants were used in 131 pregnancies (88.5%), antimalarials in 103 (69.6%), antihypertensives in 39 (26.4%), and anticoagulants in 6 (4.1%). Disease activity, medication and comparisons are shown in Table 2.

**Table 2:** Medications and SLE status before and during 155 pregnancies, along with comparisons between these periods

Characteristics	Before*, n (%)*	N$^1$	During*, n (%)*	N$^1$	$\Delta\%^2$	N$^1$	*p*
**Medications**							
Prednisone, n (%)	131 (88.5)	148	138 (91.4)	151	0.7	144	1.000
Prednisone dose, median [P25; P75]	10 [5; 20]	147	10 [5; 20]	154	0.0	147	0.647
Azathioprine, n (%)	37 (25.0)	148	35 (27.8)	126	0.0	120	1.000
Cyclophosphamide, n (%)	11 (7.4)	148	9 (7.1)	123	0.9	117	1.000
Mycophenolate, n (%)	3 (2.0)	148	4 (3.3)	126	0.0	120	1.000
Methotrexate, n (%)	2 (1.4)	148	3 (2.4)	122	0.0	116	1.000
Hydroxychloroquine, n (%)	34 (23.0)	148	57 (44.5)	128	14.0	121	0.002
Chloroquine, n (%)	69 (46.6)	148	60 (44.4)	135	-5.4	129	0.324
Angiotensin II receptor blocker, n (%)	18 (12.2)	148	17 (13.2)	129	0.0	123	1.000
Angiotensin-converting enzyme inhibitor, n (%)	18 (12.2)	148	15 (12.0)	125	-1.7	119	1.000
Calcium channel blocker, n (%)	13 (8.8)	148	17 (13.3)	128	3.3	121	0.219
Beta blocker, n (%)	8 (5.4)	148	8 (6.4)	123	0.0	119	1.000
Methyldopa, n (%)	2 (1.4)	148	34 (27.2)	125	27.1	118	*≤*0.001
Hydrochlorothiazide, n (%)	11 (7.1)	148	5 (4.1)	123	-4.1	121	0.125
Warfarin, n (%)	6 (4.1)	148	8 (6.5)	125	0.9	117	1.000
Acetylsalicylic acid, n (%)	11 (7.4)	148	31 (24.4)	127	15.8	120	*≤*0.001
Enoxaparin, n (%)	0 (0.0)	148	6 (4.9)	122	5.2	116	–
Unfractionated heparin, n (%)	0 (0.0)	148	5 (4.1)	122	3.4	116	–
Immunosuppressants, n (%)	131 (88.5)	148	139 (92.1)	151	1.4	144	0.754
Anticoagulant, n (%)	6 (4.1)	148	15 (12.2)	123	6.0	117	0.016
Antihypertensives, n (%)	39 (26.4)	148	55 (40.4)	136	11.6	129	0.001
Antimalarial, n (%)	103 (69.6)	148	106 (75.2)	141	-0.7	134	1.000
**SLE activity**							
SLEDAI, median [P25; P75]	2 [0; 6]	147	3.5 [0; 8]	154	1.5	146	*≤*0.001
Activity, n (%)	56 (38.1)	147	77 (50.0)	154	12.3	146	0.004
*≤* 3, n (%)	91 (61.9)	147	77 (50.0)	154	-12.3	146	0.004
4 – 11, n (%)	49 (33.3)	147	54 (35.1)	154	2.7	146	0.665
*≥* 12, n (%)	7 (4.8)	147	23 (14.9)	154	9.6	146	0.007
Renal, n (%)	39 (26.5)	147	54 (41.5)	130	13.8	123	0.002
Cutaneous, n (%)	30 (20.3)	148	44 (34.9)	126	9.9	121	0.050
Joint, n (%)	18 (12.2)	148	28 (21.9)	128	7.3	123	0.080
Hematological, n (%)	26 (17.7)	147	23 (19.3)	119	7.0	114	0.096
Serous, n (%)	2 (1.4)	148	1 (0.8)	118	-1.8	113	0.500
Neurological, n (%)	2 (1.4)	148	0 (0)	118	-1.7	113	–
Vasculitis, n (%)	2 (1.4)	148	1 (0.8)	122	0.9	116	1.000
Myositis, n (%)	0 (0.0)	148	3 (2.5)	122	0.8	117	–

^1^Valid N. The difference between the total N of 155 and the valid N corresponds to the missing data

^2^Corresponds to the difference in proportions or medians during the McNemar or Wilcoxon comparison tests

### Pregnancy period

In 77 (50.0%; N=154) pregnancies, patients had SLE manifestations; nephritis occurred in 54 (70.1%), cutaneous activity in 44 (57.1%), arthritis in 28 (36.6%), and hematological disorders in 23 (29.8%). The incidence of SLE activity was 29.7%; the proportion of patients with active disease increased by 12.3% compared to that in the prepregnancy period, there was also a worsening of SLEDAI (Z=-4.0; p* < *0.001); renal activity increased by 13.8%, cutaneous activity by 9.9%, and the others showed no significant change. There was an increase of 27.1% in the use of methyldopa, 15.8% in the use of ASA, and 6% in the use of anticoagulants. The other medications showed no significant variation. Table 2.

### Postpartum period

After pregnancy, SLE was in remission in 61 patients (40.9%; N=149). Among the 88 patients (59.1%) with active disease, 58 (65.9%) had nephritis, 57 (64.7%) had cutaneous activity, 33 (37.5%) had arthritis, and 14 (15.9%) had hematological activity. There was a 20.4% increase (p* < *0.001; N=142) in the proportion of SLE activity cases compared to the prepregnancy period and a worsening of the SLEDAI (4 [1; 8] vs. 2 [0; 6]; Z=-4.8; N=146; p* < *0.001). Of the patients who were in remission before pregnancy, 25 (28.7%) showed SLE flare during pregnancy, and after delivery, this proportion increased to 47.1% (p=0.005; N=87), with an incidence of 18.4%.

### Analysis of maternal and fetal/perinatal outcomes

One hundred and sixteen pregnancies (80.0%; N=145) had some maternal complication. Fifty-one pregnancies (41.5%; N=123) experienced hypertension during pregnancy. There was pregnancy loss in 35 (22.9%; N=153), preeclampsia in 26 (16.8%; N=155), SLE flare during pregnancy in 27 (18.5%; N=146), and flare after delivery in 29 patients (19.5%; N=149). In 10 pregnancies, there were no data to determine if a complication had occurred. Blood pressure data were unavailable for 6 cases (3.8%), and disease activity data were unavailable for 4 cases (2.5%).

Fetal and perinatal complications occurred in 92 pregnancies (63.9%; N=144). There were 118 live births (77.1%; N=153), of which 74 (62.7%) were term and 44 (37.3%) were premature. The median duration of pregnancy was 37 weeks [35; 39], the mean birth weight was 2772.7 grams (*±* 692.2), the median birth length was 47 cm [44.5; 49], and the median head circumference was 33 cm [32; 35]. Low birth weight for gestational age was observed in 16 cases (16.2%; N=99), and IUGR was observed in 15 cases (15.6%; N=96). There were 35 cases of fetal death (22.9%; N=153), 24 spontaneous abortions (15.6%; N=153) and 11 stillbirths (7.2%; N=153). In 11 pregnancies (7.1%), sufficient data were not available to determine if at least one fetal and/or perinatal complications had ocurred. Of these, 9 (81.8%) were related to birth weight, and in 2 cases (18.1%), no data were available at all.

Table 3 shows the comparison of the number of consultations during pregnancy, maternal age at the time of pregnancy, disease duration, and SLEDAI before and/or during pregnancy between pregnancies with and without complications. Table 4 presents the adjusted odds ratio of associations with maternal and fetal/perinatal complications for associations with $\alpha \leq 0.25$; variables showing multicollinearity were excluded from the adjustment, as shown in Tables A1 and A2 of the appendix.

**Table 3:** Comparison of clinical and demographic characteristics between SLE pregnancies with or without adverse outcomes

Characteristic	No	Yes	N$^1$	Z/t	*p*
**Maternal Events**					
Number of prenatal visits, median [P25; P75]	4 [3; 5]	3 [2;^5^]	134	1.1	0.276
Maternal age (years), mean (*±* standard deviation)	26.9 (*±*0.9)	28.5 (*±*0.5)	145	-1.3	0.178
Disease duration (months), median [P25; P75]	84 [24; 102]	72 [36; 120]	145	0.1	0.907
SLEDAI (before pregnancy), median [P25; P75]	0 [0; 1]	2 [0; 6]	141	-3.6	* < *0.001
**Fetal and Perinatal Events**					
Number of prenatal visits, median [P25; P75]	4 [3; 5]	3 [2; 5]	133	1.6	0.107
Maternal age (years), mean (*±* standard deviation)	28.1 (*±*0.8)	28.4 (*±*0.6)	155	-0.3	0.765
Disease duration (months), median [P25; P75]	60 [36; 105]	72 [36; 120]	144	-0.6	0.576
SLEDAI (before pregnancy), median [P25; P75]	0 [0; 2]	2 [0; 6]	136	-2.8	0.005
SLEDAI (during pregnancy), median [P25; P75]	0 [0; 2]	6 [0; 10]	143	-4.9	* < *0.001

^1^Valid N. The difference between the total N of 155 and the valid N corresponds to the missing data.

**Table 4:** Adjusted odds ratio of characteristics associated with adverse maternal and fetal outcomes

Characteristic			OR [IC 95%]	AOR [IC 95%]	*p*
**Maternal Events**					
Renal involvement			5.24 [1.98; 13.84]	5.58 [1.02; 30.65]	0.048
Oral ulcer			2.80 [1.06; 7.40]	2.90 [0.91; 9.20]	0.071
Immunological disorder			1.64 [0.72; 3.74]	4.18 [1.19; 14.67]	0.026
Hematological disorder			0.57 [0.25; 1.31]	0.76 [0.26; 2.21]	0.610
Anticardiolipin			0.55 [0.22; 1.36]	0.71 [0.19; 2.62]	0.603
Systemic arterial hypertension			4.92 [1.40; 17.23]	1.51 [0.25; 9.13]	0.655
Dyslipidemia			5.14 [0.66; 40.23]	2.10 [0.20; 22.58]	0.539
Chronic kidney disease			4.11 [1.34; 12.58]	0.33 [0.04; 2.53]	0.284
Angiotensin II receptor blocker			4.96 [0.63; 38.91]	0.90 [0.06; 13.97]	0.940
Hydroxychloroquine			0.32 [0.13; 0.78]	0.27 [0.09; 0.82]	0.021
Warfarin			0.24 [0.05; 1.24]	0.37 [0.05; 2.54]	0.311
Azathioprine			3.42 [0.97; 12.11]	1.50 [0.34; 6.62]	0.591
SLE activity before pregnancy			7.25 [2.07; 25.33]	3.60 [0.84; 15.44]	0.084
**Fetal and Perinatal Events**					
Renal involvement			2.36 [1.17; 4.75]	0.78 [0.17; 3.63]	0.753
Anti-double stranded DNA			2.16 [1.07; 4.34]	4.27 [1.16; 15.73]	0.029
Anti-SM			0.45 [0.14; 1.41]	0.12 [0.01; 1.38]	0.089
Systemic arterial hypertension			3.38 [1.42; 8.00]	0.41 [0.06; 2.63]	0.348
Dyslipidemia			12.41 [1.60; 95.89]	2.35 [0.19; 29.57]	0.507
Preeclampsia			2.78 [0.98; 7.89]	0.72 [0.14; 3.70]	0.693
Hypertension during pregnancy			4.41 [1.89; 10.28]	3.56 [0.83; 15.30]	0.087
Antimalarial$^1$			0.28 [0.12; 0.65]	0.08 [0.02; 0.43]	0.003
Prednisone			1.35 [0.48; 3.80]	3.78 [0.52; 27.41]	0.188
Azathioprine			1.31 [0.58; 2.97]	1.20 [0.26; 5.65]	0.814
Mycophenolate			2.27 [0.46; 11.15]	1.03 [0.02; 54.20]	0.989
Methyldopa			3.57 [1.39; 9.17]	0.34 [0.04; 2.61]	0.298
Acetylsalicylic acid			5 [1.75; 14.28]	16.48 [2.39; 113.67]	0.004
SLE activity during pregnancy			8.39 [3.77; 18.68]	22.76 [4.70; 110.14]	* < *0.001

^1^Corresponds to pregnancies in which patients were using hydroxychloroquine or chloroquineprior to pregnancy and manteined until the birth.

### Predictors of maternal, fetal, and perinatal complications

The logistic regression model for maternal complications included 141 (90.9%) pregnancies, had a significant reduction in the -2 log-likelihoods $({\chi}^2=31.88, p < 0.001)$, and had a satisfactory fit of the data according to the Hosmer and Lemeshow test $({\chi}^2=1.94, p=0.983)$; the accuracy was 80.9%. The ACR/97 criteria for immunological disorders 3.02 [1.11; 8.21], renal disorder 3.74 [1.25; 11.21], and SLEDAI 1.30 [1.04; 1.64] were risk factors, while the use of hydroxychloroquine before pregnancy was protective 0.23 [0.08; 0.67].

In the model analyzing fetal and perinatal complications, 102 pregnancies (65.8%) were included in the model. This model showed a significant reduction in the -2 Log-likelihoods $({\chi}^2=40.26, p < 0.001)$, and had a satisfactory fit of the data according to the Hosmer and Lemeshow test $({\chi}^210.38, p=0.168)$; the accuracy was 77.5%. The use of hydroxychloroquine before and during pregnancy was a protective factor 0.14 [0.04; 0.44], while the use of ASA 5.22 [1.33; 20.54] and SLEDAI 1.31 [1.14; 1.52] were risk factors for these events. Table 5 describes the regression parameters.

**Table 5:** Logistic regression models to determine risk factors for adverse events in SLE pregnancies

Characteristic	S.E.	*β*	OR	[IC 95%]	*p*
**Maternal Events** (N$^1$=141)					
Immunological alterations	0.511	1.10	3.02	[1.11; 8.21]	0.031
Renal involvement	0.560	1.32	3.74	[1.25; 11.21]	0.018
Hydroxychloroquine before pregnancy	0.535	-1.45	0.23	[0.08; 0.67]	0.007
SLEDAI before pregnancy (continuous)	0.117	0.26	1.30	[1.04; 1.64]	0.023
Constant	0.417	0.15	1.17	–	0.711
**Fetal and Perinatal Events** (N$^1$=102)					
Antimalarial prior to until the birth	0.598	-1.98	0.14	[0.04; 0.44]	0.001
Acetylsalicylic acid during pregnancy	0.698	1.65	5.22	[1.33; 20.54]	0.018
SLEDAI during pregnancy (continuous)	0.074	0.27	1.31	[1.14; 1.52]	* < *0.001
Constant	0.515	0.44	1.55	–	0.396

^1^Valid N. The difference between the total N of 155 and the valid N corresponds to themissing data.

S.E: Standard error

### Post-hoc analysis of ASA association and adverse fetal and perinatal events

Considering that ASA use is a well-established protective factor for fetal complications in pregnancy Askie et al. [20], Rolnik et al. [21], and our results unexpectedly identified it as a risk factor, we conducted a post-hoc analysis. Stratification of the association between ASA use and adverse fetal events by aPL, APS, and previous thromboembolic events (PTE) revealed a higher proportion of ASA use and fetal complications among individuals without these conditions: 13 (92.9%), 14 (87.5%) and 17 (85.0%), respectively.

The difference in proportions showed high variability, with a relative error of 26% and a wide confidence interval. Logistic regression analysis further demonstrated variability, with ASA showing a standard error of 69.8%. To address this, we applied a bootstrap analysis with 10000 resamplings, resulting in coefficient convergence and corrected standard errors. Despite these adjustments, ASA remained strongly associated with adverse fetal/perinatal outcomes. Table 6.

**Table 6:** Logistic regression model for fetal adverse events after bootstrap adjustment

Characteristic	S.E.	*β*	OR	[IC 95%]
**Fetal and Perinatal Events (N**$^1$=102)				
Antimalarial prior to until the birth	0.004	-1,37	0.25	[0.04; 0.44]
Acetylsalicylic acid during pregnancy	0.004	1.09	2.98	[1.25; 6.93]
SLEDAI during pregnancy (continuous)	0.001	0.29	1.34	[1.16; 1.65]
Constant	0.004	0.022	–	–

^1^Valid N. The difference between the total N of 155 and the valid N correspondsto the missing data.

S.E: Standard error

We then assessed the probability of sign error (Type-S) and exaggeration factor (Type-M) using the methodology of Gelman and Carlin [22] the effect size from Askie et al. [20]. The analysis revealed a 100% probability of a Type-S error and a Type-M error of -0.9996, yielding a corrected– of -1.091 and an OR of 0.33 [0.33; 0.34] for ASA association.

These findings should be interpreted with caution, as the 100% probability of a Type S error indeed supports the interpretation that aspirin acted as a protective factor, as previously discussed. The observed variability in the data suggests the potential influence of unmeasured confounding factors, such as treatment adherence, drug exposure, diagnostic access bias, or confounding by indication. However, such variables were not available in the our patient registry data.

## Discussion

The present study analyzed the incidence of adverse fetal, perinatal, and maternal events in patients with SLE in a Rheumtology outpatient clinic in the State of Amazonas and their associated risk factors.

Fetal/perinatal complications such as fetal death, prematurity, low birth weight for gestational age, and intrauterine growth restriction were frequently observed in our sample. However, the incidence distribution of these complications was not significantly different from those in the studies by Clark et al. [23], Braga et al. [24], Mokbel et al. [25].

Regarding maternal complications, the incidence of preeclampsia had a distribution similar to those found in the studies by Wu et al. [26], Clowse [27]. However, the occurrence of hypertension during pregnancy was significantly higher than the findings of Wu et al. [26]. Although this difference may correspond to an information bias, given the retrospective nature of the study, there was a significant increase in antihypertensive use during pregnancy.

The positivity of anti-double stranded DNA, aPL or anti-SM corresponds to the ACR/97 criterion for immunological disorders, and its presence increased the chance of adverse maternal events by three times. In the studies by Lima et al. [28], Buyon et al. [29], lupus anticoagulant (LAC) and anticardiolipin antibody (aCL) were predictors of adverse maternal pregnancy outcomes. Lockshin et al. [4] and Gebhart et al. [30] demonstrated that the presence of LAC increased the chance of gestational complications by 70%, and Clowse et al. [31] showed an association between the presence of anti-double stranded DNA and adverse maternal and fetal outcomes.

In our study, a history of nephritis increased the chance of maternal pregnancy outcomes by 3.7-fold. In a meta-analysis by Smyth et al. [2], this characteristic was associated with preeclampsia, and in a study by Saavedra et al. [32] it was associated with maternal complications, particularly disease activity during pregnancy, preeclampsia, and premature rupture of membranes. In the study by Mokbel et al. [25], a history of nephritis conferred a 2.3-fold greater chance of disease exacerbation during pregnancy.

The use of hydroxychloroquine before pregnancy was a protective factor against maternal complications in our sample, reducing the chance of these events by 77%. In a systematic review and meta-analysis, Hu et al. [33] reported a 26% reduction in the risk of SLE activity and a 46% reduction in the risk of preeclampsia with the use of this medication.

In our analysis, the use of ASA during pregnancy was likely influenced by various sources of bias. According to the meta-analysis conducted by Askie et al. [20], ASA use was associated with a 10% reduction in the risk of adverse fetal and perinatal outcomes. Similarly, the double-blind randomized clinical trial by Rolnik et al. [21] reported a 62% reduction in prematurity related to preeclampsia among those who used ASA. As previously discussed, these discrepancies may be attributed to data variability, unmeasured confounding variables, differences in adherence, exposure bias, diagnostic access bias, or confounding by indication.

The incidence of disease activity was lower than that observed in the studies by Urowitz et al. [34], likely reflecting scientific and therapeutic advances in recent decades, a conclusion similar to that of Gohr et al. [35]. The prevalence of activity during pregnancy was similar to the reported in the study by Clowse [27].

Disease activity increased during pregnancy compared to the period before these events. Additionally, we observed that the SLEDAI score was greater in pregnancies that expecrienced adverse fetal, perinatal, or maternal events during all evaluated periods. Our results reinforce the association between disease activity and adverse pregnancy events, as in the multivariate analysis, SLE activity increased the chance of maternal complications by 30% and fetal/perinatal complications by 31%, similar to the results described by Mokbel et al. [25], Saleh et al. [36].

## Limitations

Although the study showed results similar to those in the current literature, it has limitations inherent to its retrospective design, such as the presence of missing data, particularly regarding laboratory tests for determining SLEDAI, possibly underestimating its score and impacting the analyses.

Differentiating between preeclampsia and renal activity is a challenge, as both have similar clinical and laboratory manifestations, and the lack of laboratory data to accurately differentiate them did not allow for the specific contribution of each to adverse pregnancy events. Preeclampsia is a known risk factor for adverse pregnancy events. Although no association was found in our sample, it is necessary to consider the possibility of a type II error due to sample size being smaller than necessary to detect this specific difference.

APS is a risk factor for gestational complications, and issues related to the availability of antiphospholipid antibody tests may have interfered with the diagnosis of this comorbidity, and the overlap of the hypercoagulable state of SLE may have made it difficult to distinguish between the two conditions, leading to either an overdiagnosis or underdiagnosis of APS, possibly affecting frequency distributions and test results in a way we cannot measure.

## Conclusions

In conclusion, managing pregnancy in patients with SLE remains a challenge. Disease activity is directly related to pregnancy outcomes and should be carefully monitored before, during, and after pregnancy. Pregnancy planning is essential, and patients should be advised and encouraged to express their desire to conceive during follow-up with their rheumatologist so that SLE is controlled, contraindicated medications are discontinued or replaced before conception, and a multidisciplinary approach and monitoring, involving obstetricians experienced in high-risk pregnancies should be instituted.

## Appendix A

Tables comparing clinical characteristics, past medical history, medications, and SLE activity in pregnancies that experienced adverse maternal, fetal, or perinatal events.

**Table A1:** Comparison of characteristics among 116 pregnancies with maternal complications

Characteristic	No, *n (%)*	Yes, *n (%)*	$\Delta\%$	$N^1$	OR [IC 95%]	*p*
**ACR Criteria/Autoantibodies**						
Photosensitivity	18 (75.0)	98 (81.0)	6.0	116	1.42 [0.51; 3.98]	0.577
Malar Rash	33 (84.6)	83 (78.3)	-6.3	116	0.66 [0.25; 1.76]	0.399
Discoid Rash	107 (80.5)	9 (75.0)	-5.5	116	0.73 [0.18; 2.88]	0.707
Oral Ulcers	67 (74.4)	49 (89.1)	14.6	116	2.80 [1.06; 7.40]	0.032
Arthritis	14 (87.5)	102 (79.1)	-8.4	116	0.54 [0.12; 2.52]	0.740
Serositis	77 (77.8)	39 (84.8)	7.0	116	1.59 [0.63; 4.05]	0.326
ANA	4 (80.0)	112 (80.0)	0.0	116	1.00 [0.11; 9.30]	1.000
Immunological disorder	42 (75.0)	74 (83.1)	8.1	116	1.64 [0.72; 3.74]	0.233
Neurological disorder	106 (80.9)	10 (71.4)	-9.5	116	0.59 [0.17; 2.03]	0.480
Hematological disorder	60 (84.5)	56 (75.7)	-8.8	116	0.57 [0.25; 1.31]	0.184
Renal disorder	49 (68.1)	67 (91.8)	23.7	116	15.24 [1.98; 13.84]	< 0.001
Anti-dsDNA	58 (79.5)	58 (80.6)	1.1	116	1.07 [0.47; 2.42]	0.868
Anti-SM	104 (80.0)	12 (80.0)	0.0	116	1.00 [0.26; 3.80]	1.000
Anti-SSA	89 (79.5)	27 (81.8)	2.4	116	1.16 [0.43; 3.15]	0.766
Anti-SSB	112 (80.6)	4 (66.7)	-13.9	116	0.48 [0.08; 2.77]	0.404
Lupus Anticoagulant	108 (80.0)	8 (80.0)	0.0	116	1.00 [0.20; 4.98]	1.000
Anticardiolipin	93 (82.3)	23 (71.9)	-10.4	116	0.55 [0.22; 1.36]	0.193
Antiphospholipid	90 (81.8)	26 (74.3)	-7.5	116	0.64 [0.26; 1.58]	0.332
Anti-RNP	113 (80.1)	3 (75.0)	-5.1	116	0.74 [0.07; 7.42]	1.000
**Past Medical History**						
Chronic Kidney Disease	70 (73.7)	46 (92.0)	18.3	116	4.11 [1.34; 12.58]	0.009
Systemic Arterial Hypertension	74 (74.0)	42 (93.3)	19.3	116	4.92 [1.40; 17.23]	0.007
Dyslipidemia	98 (77.8)	18 (94.7)	17.0	116	5.14 [0.66; 40.23]	0.123
Diabetes^2^	112 (79.4)	4 (100.0)	20.6	116	–	0.584
APS	103 (80.5)	13 (76.5)	-4.0	116	0.79 [0.24; 2.63]	0.748
Aseptic Osteonecrosis	111 (80.4)	5 (71.4)	-9.0	116	0.61 [0.11; 3.31]	0.627
Previous Thromboembolic Event	106 (80.9)	10 (71.4)	-9.5	116	0.59 [0.17; 2.03]	0.480
**Medications Used Before Pregnancy**						
Prednisone	11 (68.8)	102 (81.0)	12.2	113	1.93 [0.61; 6.08]	0.321
Azathioprine	81 (75.7)	32 (91.4)	15.7	113	3.42 [0.97; 12.11]	0.054
Cyclophosphamide^2^	111 (79.3)	2 (100.0)	20.7	113	–	1.000
Mycophenolate	104 (79.4)	9 (81.8)	2.4	113	1.17 [0.24; 5.73]	1.000
Methotrexate^2^	110 (79.1)	3 (100.0)	20.9	113	–	1.000
Hydroxychloroquine	92 (84.4)	21 (63.6)	-20.8	113	0.32 [0.13; 0.78]	0.010
Chloroquine	58 (78.4)	55 (80.9)	2.5	113	1.17 [0.51; 2.65]	0.712
Angiotensin II Receptor Blocker	96 (77.4)	17 (94.4)	17.0	113	4.96 [0.63; 38.91]	0.122
Angiotensin-Converting Enzyme Inhibitor^2,3^	95 (76.6)	18 (100.0)	23.4	113	–	0.024
Calcium Channel Blocker	101 (78.3)	12 (92.3)	14.0	113	3.33 [0.41; 26.70]	0.468
Methyldopa	111 (79.3)	2 (100.0)	20.7	113	–	1.000
Hydrochlorothiazide	104 (79.4)	9 (81.8)	2.4	113	1.17 [0.24; 5.73]	1.000
Beta Blocker^2,3^	105 (78.4)	8 (100.0)	21.6	113	–	0.207
Warfarin	110 (80.9)	3 (50.0)	-30.9	113	0.24 [0.05; 1.24]	0.100
Acetylsalicylic acid	104 (79.4)	9 (81.8)	2.4	113	1.17 [0.24; 5.73]	1.000
Unfractionated Heparin^2^	–	–	–	–	–	–
Enoxaparin^2^	–	–	–	–	–	–
**Disease Activity Before Pregnancy**						
SLE activity^3^	61 (70.1)	51 (94.4)	24.3	112	7.25 [2.07; 25.33]	0.001
Renal^3^	75 (72.8)	37 (97.4)	24.6	112	13.81 [1.81; 105.51]	0.001
Cutaneous	87 (77.7)	26 (86.7)	9.0	113	1.87 [0.60; 5.86]	0.278
Articular^3^	98 (77.8)	15 (93.8)	16.0	113	4.29 [0.54; 33.87]	0.193
Hematological	99 (79.8)	13 (76.5)	-3.4	112	0.82 [0.25; 2.73]	0.752
Serosal	112 (80.0)	1 (50.0)	-30.0	113	0.25 [0.02; 4.12]	0.368
Neurological	112 (80.0)	1 (50.0)	-30.0	113	0.25 [0.02; 4.12]	0.368
Muscular	–	–	–	–	–	–
Vascular	111 (79.3)	2 (100.0)	20.7	113	–	1.000

^1^Valid N. The difference between the total N of 155 and the valid N corresponds to the missing data

^2^There were cells with zero counts

^3^Variables removed from the adjustment due to multicollinearity

**Table A2:** Comparison of characteristics among 92 pregnancies with fetal/perinatal complications

Characteristic	No, *n (%)*	Yes, *n (%)*	$\Delta\%$	$N^1$	OR [IC 95%]	*p*
**ACR/97 Criteria**						
Photosensitivity	15 (65.2)	77 (63.6)	-1.6	92	0.93 [0.37; 2.38]	0.885
Malar Rash	27 (64.3)	65 (63.7)	-0.6	92	0.98 [0.46; 2.06]	0.949
Discoid Rash	84 (63.2)	8 (72.7)	9.5	92	1.56 [0.39; 6.14]	0.746
Oral Ulcers	57 (60.6)	35 (70.0)	9.4	92	1.51 [0.73; 3.15]	0.265
Arthritis	11 (68.8)	81 (63.3)	-5.5	92	0.78 [0.26; 2.39]	0.668
Serositis	62 (63.9)	30 (63.8)	-0.1	92	1.00 [0.48; 2.06]	0.999
ANA	3 (75.0)	89 (63.6)	-11.4	92	0.58 [0.06; 5.74]	1.000
Immunological disorder	35 (60.3)	57 (66.3)	6.0	92	1.29 [0.65; 2.58]	0.467
Neurological disorder	81 (62.8)	11 (73.3)	10.5	92	1.63 [0.49; 5.40]	0.421
Hematological disorder	45 (66.2)	47 (61.8)	-4.4	92	0.83 [0.42; 1.64]	0.589
Renal disorder	39 (54.2)	53 (73.6)	19.4	92	2.36 [1.17; 4.75]	0.015
**Autoantibody Profile**						
Anti-double stranded DNA	41 (55.4)	51 (72.9)	17.5	92	2.16 [1.07; 4.34]	0.029
Anti-SM	86 (65.6)	6 (46.2)	-19.4	92	0.45 [0.14; 1.41]	0.226
Anti-SSA	71 (65.7)	21 (58.3)	-7.4	92	0.73 [0.34; 1.58]	0.423
Anti-SSB	88 (64.2)	4 (57.1)	-7.1	92	0.74 [0.16; 3.45]	0.703
Lupus Anticoagulant	85 (63.4)	7 (70.0)	6.6	92	1.35 [0.33; 5.44]	1.000
Anticardiolipin	72 (64.9)	20 (60.6)	-4.3	92	0.83 [0.37; 1.85]	0.655
Antiphospholipid	70 (64.2)	22 (62.9)	-1.3	92	0.94 [0.43; 2.08]	0.884
Anti-RNP	90 (64.3)	2 (50.0)	-14.3	92	0.56 [0.08; 4.07]	0.620
**Maternal Comorbidities**						
Chronic Kidney Disease^2^	52 (54.2)	40 (83.3)	29.1	92	4.23 [1.79; 9.99]	* < *0.001
Systemic Arterial Hypertension	57 (56.4)	35 (81.4)	25.0	92	3.38 [1.42; 8.00]	0.004
Dyslipidemia	74 (59.2)	18 (94.7)	35.5	92	12.41 [1.60; 95.89]	0.003
Diabetes^3^	89 (63.1)	3 (100.0)	36.9	92	–	0.553
APS	79 (62.7)	13 (72.2)	9.5	92	1.55 [0.52; 4.61]	0.431
Aseptic Osteonecrosis	87 (63.5)	5 (71.4)	7.9	92	1.44 [0.27; 7.68]	1.000
Previous Thromboembolic Event	82 (64.1)	10 (62.5)	-1.6	92	0.93 [0.32; 2.74]	0.902
**Pregnancy Complications**						
Preeclampsia	71 (60.2)	21 (80.8)	20.6	92	2.78 [0.98; 7.89]	0.048
Hypertension during pregnancy	31 (46.3)	38 (79.2)	32.9	69	4.41 [1.89; 10.28]	* < *0.001
**Medications Used Before Pregnancy**						
Prednisone	10 (58.8)	79 (65.8)	7.0	89	1.35 [0.48; 3.80]	0.572
Azathioprine	64 (63.4)	25 (69.4)	6.0	89	1.31 [0.58; 2.97]	0.512
Cyclophosphamide^3^	87 (64.4)	2 (100.0)	35.6	89	–	0.541
Mycophenolate	81 (63.8)	8 (80.0)	16.2	89	2.27 [0.46; 11.15]	0.493
Methotrexate	88 (65.7)	1 (33.3)	-32.4	89	0.26 [0.02; 2.96]	0.281
Hydroxychloroquine^2^	75 (72.1)	14 (42.4)	-29.7	89	0.28 [0.13; 0.64]	0.002
Chloroquine	46 (62.2)	43 (68.3)	6.1	89	1.31 [0.64; 2.66]	0.456
Angiotensin II Receptor Blocker^2^	75 (62.5)	14 (82.4)	19.9	89	2.80 [0.76; 10.28]	0.173
Angiotensin-Converting Enzyme Inhibitor^2^	72 (60.5)	17 (94.4)	33.9	89	11.10 [1.43; 86.20]	0.003
Calcium Channel Blocker^2^	79 (63.2)	10 (83.3)	20.1	89	2.91 [0.61; 13.87]	0.214
Methyldopa	88 (65.2)	1 (50.0)	-15.2	89	0.53 [0.03; 8.73]	1.000
Hydrochlorothiazide	82 (63.1)	8 (72.7)	9.6	90	1.56 [0.40; 6.17]	0.746
Beta Blocker	82 (63.6)	7 (87.5)	23.9	89	4.01 [0.48; 33.62]	0.260
Warfarin	86 (65.6)	3 (50.0)	-15.6	89	0.52 [0.10; 2.70]	0.422
Acetylsalicylic acid^2^	80 (63.0)	9 (90.0)	27.0	89	5.29 [0.65; 43.06]	0.165
Unfractionated Heparin^3^	–	–	–	–	–	–
Enoxaparin^3^	–	–	–	–	–	–
Antimalarial^4^	39 (81.2)	50 (54.9)	-26.3	89	0.28 [0.12; 0.65]	0.002
**Disease activity before pregnancy**						
SLE Activity^2^	46 (55.4)	42 (79.2)	23.8	88	3.07 [1.39; 6.78]	0.005
Renal^2^	58 (58.6)	30 (81.1)	22.5	88	3.03 [1.21; 7.56]	0.015
Cutaneous^2^	68 (62.4)	21 (75.0)	12.6	89	1.81 [0.71; 4.63]	0.212
Articular^2^	75 (62.5)	14 (82.4)	19.9	89	2.80 [0.76; 10.28]	0.173
Hematologic	80 (66.1)	8 (53.3)	-12.8	88	0.59 [0.20; 1.73]	0.393
Serous	88 (65.2)	1 (50.0)	-15.2	89	0.53 [0.03; 8.73]	1.000
Neurological^3^	88 (65.2)	1 (50.0)	-15.2	89	0.53 [0.03; 8.73]	1.000
Muscular^3^	–	–	–	–	–	–
Vascular^3^	87 (64.4)	2 (100.0)	35.6	89	–	0.541
**Medications during pregnancy**						
Prednisone	5 (38.5)	84 (66.1)	27.6	89	3.13 [0.96; 10.13]	0.068
Azathioprine	44 (54.3)	23 (67.6)	13.3	67	1.76 [0.76; 4.08]	0.186
Cyclophosphamide^3^	61 (56.0)	3 (100.0)	44.0	64	–	0.259
Mycophenolate	59 (55.7)	8 (88.9)	33.2	67	6.37 [0.77; 52.77]	0.078
Methotrexate	61 (57.0)	2 (50.0)	-7.0	63	0.75 [0.10; 5.56]	1.000
Hydroxychloroquine^2^	43 (67.2)	25 (47.2)	-20.0	68	0.44 [0.21; 0.92]	0.028
Chloroquine	40 (58.0)	33 (60.0)	2.0	73	1.09 [0.53; 2.24]	0.820
Angiotensin II receptor blocker^2^	56 (55.4)	14 (82.4)	27.0	70	3.75 [1.01; 13.86]	0.037
Angiotensin-converting enzyme inhibitor^2^	54 (54.0)	12 (85.7)	31.7	66	5.11 [1.09; 24.03]	0.024
Calcium channel blocker^2^	54 (54.0)	15 (88.2)	34.2	69	6.39 [1.39; 29.41]	0.008
Methyldopa	41 (50.0)	25 (78.1)	28.1	66	3.57 [1.39; 9.17]	0.006
Hydrochlorothiazide^3^	61 (57.0)	3 (60.0)	3.0	64	1.13 [0.18; 7.05]	1.000
Beta-blocker^2,3^	58 (54.7)	8 (100.0)	45.3	66	–	0.020
Warfarin	60 (57.7)	4 (50.0)	-7.7	64	0.73 [0.17; 3.09]	0.723
Acetylsalicylic acid	43 (50.0)	25 (83.3)	33.3	68	5.00 [1.75; 14.28]	0.001
Unfractionated heparin	60 (56.6)	3 (60.0)	3.4	63	1.15 [0.18; 7.17]	1.000
Enoxaparin	60 (57.1)	3 (50.0)	-7.1	63	0.75 [0.14; 3.89]	1.000
**Disease activity during pregnancy**						
SLE Activity^2^	28 (40.6)	63 (85.1)	44.5	91	8.39 [3.77; 18.68]	* < *0.001
Renal^2^	29 (42.6)	46 (88.5)	45.9	75	10.31 [3.88; 27.39]	* < *0.001
Cutaneous^2^	37 (50.0)	31 (75.6)	25.6	68	3.10 [1.33; 7.22]	0.001
Articular^2^	48 (52.7)	23 (85.2)	32.5	71	5.15 [1.65; 16.09]	0.002
Hematologic^2^	48 (55.2)	16 (72.7)	17.5	64	2.17 [0.77; 6.06]	0.135
Serous^3^	61 (57.0)	1 (100.0)	43.0	62	–	1.000
Neurological^3^	–	–	–	–	–	–
Muscular^3^	62 (56.4)	1 (100.0)	43.6	63	–	1.000
Vascular^3^	60 (55.6)	3 (100.0)	44.4	63	–	0.257

^1^The difference between the total N of 92 and the valid N corresponds to missing data

^2^Variables removed from adjustment due to multicollinearity

^3^There were cells with zero counts

^3^4Corresponds to pregnancies where patients were using hydroxychloroquine or chloroquine before pregnancy and continued until the last consultation

## Data Availability

Not applicable.
